# Whole-Genome Sequences of Two Rare Human Group C Rotavirus Strains Isolated from Two Cases of Acute Gastroenteritis

**DOI:** 10.1128/genomeA.01014-15

**Published:** 2015-09-10

**Authors:** Long Chen, De-Jian Zhao, Hong Yang, Hai-Long Zhang, Xiang-Jie Yao, Ren-Li Zhang, Ya-Qing He

**Affiliations:** a Major Infectious Disease Control Key Laboratory and Shenzhen Public Service Platform of Pathogenic Microorganisms Repository, Shenzhen Center for Disease Control and Prevention, Shenzhen, Guangdong, People’s Republic of China; b Yuexiu District Center for Disease Control and Prevention, Guangzhou, Guangdong, People’s Republic of China

## Abstract

This is a report of the complete genomic sequences of two rare group C rotavirus strains RVC/SZ94/CHN/2011 and RVC/SZ272/CHN/2011, isolated from two cases of acute gastroenteritis in Shenzhen, southern China, in 2011. These two strains display a close genetic relationship to 2007 Chinese strain YNR001 and 2008 Japanese strain BK0830.

## GENOME ANNOUNCEMENT

Rotavirus (RV), a member of the family *Reoviridae*, is the most important viral agent that causes gastroenteritis in young humans and animals (1, 2). The RV particles consist of a triple-layered capsid that surrounds a double-strand RNA genome made up of 11 segments. Each of the 11 segments contains evolutionarily conserved 5′- and 3′-terminal noncoding regions. These RNA segments encode six structural (VP1 to VP4, VP6, VP7) and five nonstructural proteins (NSP1 to NSP5). Based on the antigenicity of the inner capsid protein VP6 and genomic characteristics, RV was classified into seven groups, RV-(A to G) (3, 4). So far, RV-A, RV-B, and RV-C have been detected in humans. Human RV-A is the most prevalent in diarrheal diseases, whereas the prevalence of RV-B and RV-C is relatively low (2–4). As of now, only seven whole-genome sequences of human RV-C, not including the two genome sequences of this study, are available in the public databases.

Only two specimens (2/983, 0.2%) from hospital-based surveillance for diarrheal diseases were detected positive for human RV-C by using a real-time reverse transcription (RT)-PCR assay in Shenzhen, southern China, during 2011. We used a total of 50 sets of primers to amplify the full genomic sequences of these two strains as described previously (5). PCR products were purified using a commercial kit (catalog no. D823A, MiniBEST agarose gel DNA extraction kit v3.0, TaKaRa, Japan) and sequenced by the ABI PrismTM 3730xl DNA analyzer using the BigDye Terminator v3.1 cycle sequencing kit (Applied Biosystems). Contigs were assembled using Lasergene v7.1. The raw sequences were edited using BioEdit v7.2.5 before submission to GenBank. Nucleic acid and amino acid sequence alignments were done with BioEdit v7.2.5.

The sizes of full length segments VP1-4, VP6-7, and NSP1-5 are 3,309, 2,736, 2,166, 2,283, 1,353, 1,063, 1,270, 1,037, 1,350, 613, and 730 bp and the open reading frames (ORFs) for these segments are 3,273, 2,655, 2,082, 2,235, 1,188, 999, 1,185, 939,1,209, 453, and 639 bp, respectively. Ten genes (VP1, VP2, VP3, VP4, VP6, VP7, NSP1, NSP2, NSP3, and NSP4) of strain RVC/SZ94/CHN/2011 are closely related to the 2007 Chinese strain YNR001 (>98.1% identity) and 2008 Japanese strain BK0830 (>96.0% identity), while the NSP5 gene was most similar to the 2003 Japanese strain OH567 (99.8% identity). All genes of strain RVC/SZ272/CHN/2011 display a close genetic relationship with the 2008 Japanese strain BK0830 (>98.8% identity) and 2007 Chinese strain YNR001 (>96.4% identity). And >96.1% nucleotide identity is observed between corresponding genes of RVC/SZ94/CHN/2011 and RVC/SZ272/CHN/2011. We find that degree of variation of the outer capsid protein VP4 gene is relatively larger than that of the other genes.

A nucleotide sequence-based, complete genome classification system was developed for group A rotaviruses by the Rotavirus Classification Working Group (RCWG) (6). Now, genotyping methods for other RV groups are urgently needed to help expand our knowledge of the genetic diversity of rotaviruses.

### Nucleotide sequence accession numbers. 

The full-genome sequences of two rare human group C rotavirus strains RVC/SZ94/CHN/2011 and RVC/SZ272/CHN/2011 have been deposited in GenBank under accession no. KP342024 to KP342045.
